# Legal protection of personal health information in medical AI applications in China: challenges and regulatory responses

**DOI:** 10.3389/fpubh.2026.1855457

**Published:** 2026-08-12

**Authors:** Longmei Tian, Ruohua Ning

**Affiliations:** 1 School of Law, Tongji University, Shanghai, China; 2 Business School, Xianda College of Economics and Humanities Shanghai International Studies University, Shanghai, China

**Keywords:** China, legal protection, medical artificial intelligence, personal health information, regulation

## Abstract

Medical artificial intelligence applies advanced AI technologies to enhance the efficiency and quality of clinical diagnosis and treatment. The extensive processing of personal health information in medical AI applications have further accelerated the digital transformation of healthcare services. However, medical AI also introduces new challenges for personal health information protection. In this context, health information faces not only continuous processing but also uncertain processing purposes, involvement of multiple processing entities, and accumulating algorithmic risks, all of which may weaken individuals’ control over their health information and increase the risk of privacy infringement. Although China has established a preliminary legal framework for personal information protection, existing rules remain fragmented and insufficiently targeted. Specifically, the concept and scope of personal health information remain insufficiently defined, the legal bases for its processing require further clarification, and traditional informed consent rules are difficult to apply effectively in medical AI environments. Against this background, this article argues for the enactment of dedicated legislation on personal health information protection to establish a more systematic regulatory framework for defining health information and governing its processing. With respect to informed consent, the existing uniform requirement of separate consent should be refined through a tiered consent framework based on information classification and contextual risk assessment, complemented by dynamic consent mechanisms to enhance individuals’ continuous control over their health information throughout the data-processing lifecycle. Furthermore, in response to emerging risks arising from the application of medical AI algorithms, a lifecycle governance framework covering algorithm design, training, and deployment should be established to enhance the accountability and safety of algorithmic processing.

## 1 Introduction

Medical artificial intelligence (medical AI) refers to the application of artificial intelligence technologies, including big data analytics, machine learning, and related computational techniques, in the healthcare sector and public health system to provide personalized medical services and improve the prevention, diagnosis, treatment, and management of diseases (1, 2). In recent years, China has actively promoted the development of medical AI while simultaneously strengthening institutional safeguards for the protection of personal health information. The *Healthy China 2030 Plan Outline*, adopted in 2016, highlighted the importance of developing healthcare big data applications, strengthening medical information security, improving encryption and access control mechanisms, and establishing mechanisms for monitoring information leakage risks, thereby laying an initial foundation for personal health information governance in China’s digital healthcare transformation. More recently, China has introduced regulatory measures, including the *Interim Measures for the Administration of Generative Artificial Intelligence Services* (3) and the *Technical Requirements for the Application of Large Models in the Healthcare Industry* (4), to promote the standardized development of healthcare AI applications.

Personal health information constitutes a fundamental resource for medical AI development, as AI systems rely extensively on the collection, integration, and analysis of health-related data. Under China’s *Data Security Law and Personal Information Protection Law* (PIPL), data generally refers to records of information in electronic or other forms, while personal information refers to information relating to an identified or identifiable natural person. In the context of healthcare, health data represents the digitalized form through which health-related information is collected, stored, and processed, whereas personal health information emphasizes the connection between such information and identifiable individuals. Therefore, the development and application of medical AI, which increasingly depends on the large-scale processing of health data, are closely intertwined with the protection of personal health information.

However, the application of medical AI has significantly reshaped traditional models of health information collection and use (5). Unlike conventional healthcare practices, where health information is primarily collected and used within a relatively stable doctor–patient relationship for direct clinical purposes, medical AI systems often require large-scale data aggregation and cross-institutional sharing for model development and optimization. As a result, personal health information may circulate among multiple actors and be processed for purposes beyond the original context of collection, creating significant challenges for traditional principles of personal information protection, particularly informed consent, purpose limitation, and transparency. These challenges have already emerged in practice. In the *Google DeepMind and Royal Free London NHS Foundation Trust* case, the NHS trust transferred approximately 1.6 million patient records to DeepMind for the development of an artificial intelligence application for detecting acute kidney injury. The UK Information Commissioner’s Office subsequently found that the data processing lacked sufficient transparency because patients were not adequately informed about how their health information would be used and no effective mechanism was provided for patients to exercise meaningful control over their data (6). Similarly, in *Dinerstein v. Google* in the United States, the University of Chicago Medical Center provided Google with years of de-identified patient records to train an algorithm designed to predict patients’ future healthcare needs (7). Although the Seventh Circuit dismissed the claim due to the absence of sufficiently concrete harm, the case raised broader concerns regarding whether patients could reasonably expect their health information to be transferred to technology companies and used for AI development beyond their original healthcare context. Taken together, these cases demonstrate the persistent tension between the data-intensive development of medical AI and the protection of personal health information. To further strengthen the protection of personal health information and safeguard the legitimate rights and interests of data subjects, it is necessary to identify the challenges arising from the development of medical AI and formulate targeted regulatory measures to address these emerging issues.

Nevertheless, existing legislation and scholarly literature have primarily focused on the general governance of artificial intelligence or the broader protection of personal information, with insufficient attention to the particular nature of personal health information and the application of general personal information protection rules in medical AI contexts (8–10). Unlike ordinary personal information processing, medical AI relies on the extensive and continuous use of highly sensitive health information, creating regulatory challenges that cannot be fully addressed by general personal information protection rules. Although existing frameworks provide enhanced protection for sensitive information, they remain largely general and principle-based and lack mechanisms tailored to the distinctive characteristics of medical AI applications. Therefore, further research is needed to develop a more targeted governance framework for personal health information protection in the medical AI context.

Against this background, this article adopts a doctrinal legal research approach supplemented by comparative legal analysis. It examines China’s existing legal and regulatory framework, including the Personal Information Protection Law and related regulations, to identify structural limitations and practical shortcomings in the governance of personal health information in medical AI contexts. Comparative legal analysis of regulatory approaches in jurisdictions such as the European Union and the United States, particularly the *General Data Protection Regulation* (GDPR) and *Health Insurance Portability and Accountability Act* (HIPAA), is incorporated to provide insights for improving China’s regulatory framework. Building on these analyses, this article proposes a more targeted governance approach, beginning with the establishment of dedicated legislation on personal health information protection, followed by the optimization of consent mechanisms and the development of whole-process algorithmic governance mechanisms for medical AI applications. Through these proposals, this article seeks to contribute to a regulatory framework that balances the protection of individuals’ rights and interests in personal health information with the responsible development of medical AI.

## 2 Defining the concept and scope of personal health information

Personal health information constitutes a core component of personal information, and a clear definition of the concept is essential to the accurate delineation of its scope. In the United States, HIPAA refers to such information as “Protected Health Information” (PHI), namely individually identifiable health information relating to an individual’s past, present, or future physical or mental health condition, the provision of healthcare, or payment for healthcare. In the European Union, Article 4 (15) of the GDPR defines “data concerning health” as personal data related to the physical or mental health of a natural person, including the provision of healthcare services, which reveal information about that person’s health status (12). It follows that, under the GDPR, data concerning health is not limited to direct medical information such as medical records or diagnostic records. Pursuant to Recital 35 of the GDPR and relevant case law of the Court of Justice of the European Union, it may also include data that is not medical in nature at first glance but is capable, either independently or in combination with other data, of revealing the health status of an identified or identifiable individual (13).

In China, neither legislation nor academic scholarship has reached a settled consensus on the definition or terminology of personal health information, and the terms “health information” and “health data” are often used interchangeably. The *Civil Code* introduced the term “health information” and brought it within the broader category of personal information, but did not provide a detailed definition of its meaning or scope. The PIPL employs the term “medical and health information” and classifies it as a category of sensitive personal information, yet likewise stops short of offering a precise definitional account. There are numerous provisions in relevant industry standards and guidelines that regulate the processing of personal health information. For example, the standard *Health informatics—Guidelines on data protection to facilitate the trans-border flows of personal health information* uses the term “personal health information” and defines it as personal data concerning the health status of an identified or identifiable natural person (14). However, most of the terms used are not “personal health information” but rather “personal health data” or “personal medical health data,” though their defined meanings substantially overlap with that of personal health information. *Information security technology—Guidelines for health and medical data security* defines “personal health and medical data” as electronic data that, either alone or in combination with other information, can identify a specific natural person or reflect that person’s physical or mental health status (15).

Scholarly views likewise diverge. Some scholars define personal health information as encompassing information concerning an individual’s physical characteristics, health status, genetic information, social contacts, medical history, and medical records (11). Others argue that the scope of personal health information is broader, extending not only to the data subject’s physical and mental health status and details of medical diagnosis and treatment, but also to basic personal information and economic background information associated with medical services (16). Still others maintain that personal health information includes all information capable of identifying an individual’s health status (17). Despite these differences in emphasis, existing scholarship generally converges on two core elements: identifiability and health relevance. To facilitate the following analysis, this article defines personal health information as personal information that, either alone or in combination with other information, can identify a specific natural person and is closely related to that person’s physical or mental health, including medical information, health-monitoring information, and health-prediction information.

## 3 The current legislative status of personal health information protection in China

China has developed a multi-level legal framework for the protection of personal health information, consisting of general laws, special laws, and normative documents. First, at the level of general legislation, the foundational legal framework for personal information protection in China includes the *Civil Code*, the PIPL, and the *Data Security Law*. Together, these laws establish the basic principles and general legal bases governing the protection and processing of personal health information. Second, at the level of special legislation, sector-specific statutes in the field of healthcare, such as the *Law on the Prevention and Treatment of Infectious Diseases* and the *Law on Practicing Physicians*, impose more tailored obligations and liabilities with respect to the collection, use, storage, and confidentiality of health information by medical institutions and healthcare professionals. In terms of applicability, such special norms take precedence over general laws within their respective regulatory scope. Third, at the level of normative documents, administrative authorities responsible for health governance formulate relevant administrative measures, technical standards, and operational guidelines based on higher-level laws, thereby further specifying the procedural requirements, security standards, and practical approaches for the processing of health information. Although such normative documents rank below laws and administrative regulations in the legal hierarchy, they serve an important supplementary institutional function in administrative regulation and sectoral governance (see Table 1).

**Table 1:** Main Chinese legal instruments relevant to the protection of personal health information.

Regulatory level	Legal/regulatory instrument	Key relevance
General law	*Civil Code of the People’s Republic of China*	Article 1034: The personal information of natural persons is protected by law. Personal information refers to all kinds of information, recorded electronically or by other means, that can identify a specific natural person, either independently or in combination with other information, including … health information. For private information contained in personal information, the relevant provisions on the right to privacy shall apply; where no such provisions exist, the relevant provisions on the protection of personal information shall apply.
	*Personal Information Protection Law of the People’s Republic of China*	Article 28: Sensitive personal information refers to personal information that, once leaked or illegally used, is likely to infringe upon the personal dignity of a natural person or endanger personal or property safety, including … medical and health information … Article 29: The processing of sensitive personal information shall be subject to the individual’s separate consent.
	*Data Security Law of the People’s Republic of China*	Article 21 lays the legal foundation for establishing a system of classified and graded data protection and for strengthening protection of important data. On this basis, standards and rules have gradually been formulated across different industries and sectors to further implement classified and graded data protection, including in the healthcare sector.
	*Criminal Law of the People’s Republic of China*	Chapter IV of the Specific Provisions, “Crimes of Infringing upon Citizens’ Personal Rights and Democratic Rights,” provides for the crime of infringing citizens’ personal information.
	*Cybersecurity Law of the People’s Republic of China*	Article 41 provides that the collection of personal information must comply with the principles of legality, legitimacy, and necessity, as well as the rule of informed consent.
Special law	*Law of the People’s Republic of China on Practicing Physicians*	Article 56 expressly provides for the scope of physicians’ duty of confidentiality, the exceptions thereto, and the legal liabilities arising from breach of that duty.
	*Law of the People’s Republic of China on the Prevention and Treatment of Infectious Diseases*	Article 12 authorizes the collection of necessary personal information for the purposes of epidemic prevention and control, while at the same time prohibiting its disclosure.
Normative document	*Provisions on the Management of Medical Records in Medical Institutions*	Article 6 requires medical institutions to establish a confidentiality system for medical records. It strictly prohibits anyone from disclosing, altering, damaging, selling, or illegally providing patients’ medical information.
	*Administrative Measures for Internet-based Diagnosis and Treatment (Trial)*	Article 20: Medical institutions shall strictly comply with relevant laws and regulations on information security and the confidentiality of medical data, properly preserve patient information, and shall not illegally trade in or disclose patient information. Where patient information or medical data is leaked, the medical institution shall promptly report the matter to the competent health authority and immediately take effective response measures.

Although China has established a basic legal framework for the protection of personal health information, the existing legislative approach has revealed significant limitations in addressing the challenges arising from the rapid development of medical AI. One particularly prominent deficiency is the absence of dedicated legislation specifically addressing the protection of personal health information, with the result that the relevant norms remain fragmented, abstract, and insufficiently adapted to specific application scenarios. First, under the current legal and regulatory framework, personal health information is broadly subsumed within the general framework of personal information protection. Although existing legislation has made clear the need to strengthen the protection of sensitive information, the protection of sensitive data such as personal health data still requires further refinement in specific industries and application scenarios. In particular, in an era of rapid AI development, establishing a comprehensive legal regime for the protection of personal health information in AI contexts remains a major challenge. Second, relevant medical laws and regulations mainly impose duties of confidentiality and professional responsibility on medical institutions and healthcare professionals, and their regulatory focus remains largely confined to the traditional doctor–patient relationship. As a result, they are ill-equipped to cover the diverse actors involved in the processing of health information in medical AI settings, such as algorithm developers, platform operators, cloud service providers, and other third-party processors. By contrast, although legal norms in the field of network and information regulation have relatively broad general applicability, their provisions are largely principle-based and have not yet established a more targeted regulatory framework for health information as a highly sensitive category of data or for the specific risks arising in medical contexts.

## 4 Challenges in the protection of personal health information in medical AI applications

### 4.1 Medical AI intensifies the tension between personal health information protection and use

Balancing the protection and use of personal health information has become a central challenge in the digital age (18). This tension arises primarily from the dual nature of personal health information, which embodies both private interests and broader public values. On the one hand, personal health information has a strong private character due to the sensitivity of its content and its close connection with individual privacy. Its protection primarily aims to prevent unauthorized access, disclosure, misuse, and inference of individuals’ private information. Accordingly, individuals reasonably expect stronger safeguards against the misuse and infringement of their health information. On the other hand, personal health information may also acquire a public dimension under certain conditions (19). When incorporated into broader health datasets, such information can be processed to serve wider social purposes, thereby implicating the interests of multiple stakeholders and broader public interests. This functional value gives personal health information an important public dimension, as its appropriate use can contribute not only to the efficient allocation of healthcare resources and the improvement of healthcare quality but also to clinical knowledge development, pharmaceutical research, and evidence-based public health decision-making.

Overemphasizing the use of personal health information at the expense of its protection may cause irreparable harm to patients and may also intensify conflicts between patients and healthcare providers. Conversely, giving information subjects overly absolute control over all forms of personal health information may unduly restrict its legitimate use, thereby undermining the broader goal of making AI-enabled healthcare more accessible. The widespread use of medical AI has made this tension even more pronounced. Given the heavy dependence of AI development on large-scale personal medical data for training and optimization, strong incentives arise to expand the use of personal health information. Information processors are thus increasingly driven to maximize the value of such data, creating pressure on the level of protection that information subjects may expect. Moreover, the risks generated by medical AI are no longer confined to traditional forms of harm such as data leakage. They increasingly manifest in new forms, including excessive collection, secondary use without the data subject’s consent, and algorithmic discrimination. Because these risks extend beyond conventional tort paradigms, they pose deeper challenges to the protection of personal health information and render the balance between protection and use increasingly difficult to sustain (20).

### 4.2 The limited effectiveness of purpose limitation and informed consent in medical AI settings

As a foundational principle of personal information protection, purpose limitation applies throughout the entire lifecycle of information processing and imposes generally binding requirements on all medical data processing activities. Its core requirement is that medical data must be collected for specific, explicit, and lawful purposes, and that any subsequent processing must remain within a scope compatible with those original purposes. This requirement is expressly reflected in Article 6(1) of the PIPL, which further provides that personal information processing must have a clear and reasonable purpose, be directly related to that purpose, and be conducted by means that have the least possible impact on personal rights and interests. The principle of informed consent has both legal and ethical roots in the *Nuremberg Code*, which established the requirement that human subjects give voluntary consent. It subsequently developed into a core principle of bioethics and a widely accepted ethical standard for protecting the rights of research participants within the global medical community. Today, informed consent has been extended into the field of personal information protection, where it functions as a central legal basis for the processing of personal information. In terms of its constituent elements, informed consent requires both adequate disclosure and voluntary consent. The former requires healthcare providers or researchers to provide data subjects with full and accurate information about key matters, including the purpose, scope, method, and risks of data processing, so that they can make a meaningful and informed decision. The latter emphasizes the data subject’s autonomous right to give or withhold consent, as well as the legal capacity required to give valid consent. Accordingly, informed consent has evolved from a basic requirement of medical practice into a statutory principle governing personal information processing. Article 14 of the PIPL, which requires consent to be given voluntarily and explicitly on the basis of adequate knowledge, together with the purpose limitation requirement under Article 6(1), forms China’s basic normative framework for personal information processing.

Personal health information requires particular attention because it is expressly classified as sensitive personal information under the PIPL. Given its close connection with an individual’s life, health, and safety, as well as its heightened sensitivity and risk of leakage or misuse, the law imposes stricter protection standards for its processing. Under Article 28(2) of the PIPL, the processing of sensitive personal information, including medical health information, must satisfy three core requirements: a specific purpose, sufficient necessity, and enhanced protective measures. First, the processor must clearly define the specific processing purpose in advance and may not rely on vague or open-ended formulations. Second, the processing must be strictly limited to what is necessary to achieve the stated purpose and must not exceed reasonable limits. Third, before processing such information, the processor must adopt security measures stricter than those required for ordinary personal information in order to prevent risks such as data leakage, misuse, and unauthorized access. On this basis, Article 29 of the PIPL further establishes “separate consent” as a specific consent requirement for processing sensitive personal information. Compared with the ordinary consent requirement under Article 13(1), separate consent imposes a higher standard of explicitness and specificity, requiring the data subject to make a specific and unambiguous expression of intention regarding the processing of sensitive personal information. Therefore, when processing personal health information, medical data processors, including developers of medical artificial intelligence systems, must comply not only with the general principles of personal information protection but also with the special rules applicable to sensitive personal information. They must satisfy more stringent statutory requirements concerning the legal basis for processing, the necessity of the processing purpose, and the conditions for valid consent.

However, in the context of medical AI, the principles of purpose limitation and informed consent are often difficult to operationalize in practice. Unlike information processing in traditional diagnosis and treatment, where purposes and contexts are relatively fixed, medical AI relies on continuous, large-scale, and cross-contextual data flows. Data sources have expanded beyond hospital records to include a wide range of sources, such as wearable devices and health applications. As processing chains become longer and the purposes of algorithm training continue to evolve, individuals are often unable, at the time of authorization, to understand who will use their information, how it will be processed, which models it will support, and for what subsequent purposes it may be reused. Moreover, medical AI products and services typically obtain consent through bundled privacy policies, yet such authorization often fails to distinguish clearly among the uses, sharing arrangements, and risk implications associated with different categories of health information. Because medical services and health-management tools are often practically indispensable, individuals rarely enjoy equal bargaining power or a genuine ability to refuse. Their purported consent therefore more closely resembles passive acceptance under conditions of information asymmetry and service dependence (21). It should be acknowledged that this problem is not unique to AI-enabled processing of personal health information. More fundamentally, it reflects a broader structural limitation of informed consent in healthcare settings, where professional authority, medical knowledge asymmetry, communication quality, and patients’ dependence on medical expertise may place patients in a relatively passive position (22). In the context of medical AI, these constraints are further intensified, as patients may formally authorize data use without fully understanding how their information will be processed, shared, reused, or incorporated into algorithmic systems.

A parallel difficulty arises from the side of information processors. Large-scale algorithmic applications typically integrate multiple techniques and complex parameter settings. After repeated modification and updating, the logic of the system often becomes extremely difficult to explain, making compliance with disclosure obligations highly challenging in practice (23). Besides, requiring separate consent for every instance of data access or model optimization would not only impose substantial coordination and compliance costs, but also significantly slow technological iteration and research. Yet relying on an initial blanket authorization to cover all possible future uses of data risks reducing informed consent to a purely formal compliance mechanism. In this sense, medical AI creates a structural dilemma for informed consent: strict adherence to individualized consent requirements may be operationally unworkable, whereas reliance on generalized consent undermines the substantive protective function of consent itself. Under these conditions, informed consent is significantly weakened in practice. Although it remains, at the normative level, an important legal basis for the processing of health information, it may become little more than a procedural formality through which information processors shift risk and responsibility (24). As a result, the legally recognized rights to informed consent and control over personal health information face serious obstacles to meaningful realization in medical AI settings.

### 4.3 Algorithmic risks in medical AI complicate the protection of personal health information

Algorithmic bias may further aggravate the risk of discrimination in the processing of personal health information. The performance of medical artificial intelligence models depends heavily on the quality and representativeness of training data, as well as on the guiding values embedded in the design of the models themselves (25). Where the underlying data exhibit structural imbalances with respect to gender, age, region, disease distribution, or socioeconomic status, or where the model adopts biased indicators as prediction targets, the algorithm may reproduce or even amplify existing social inequalities during training and deployment while concealing its discriminatory consequences behind a veneer of “technological neutrality” (26). For example, some groups may have their actual health risks systematically underestimated by models because of historically limited access to medical resources, incomplete treatment records, or chronically insufficient data samples, thereby placing them at a disadvantage in risk assessment and the allocation of diagnostic and therapeutic resources. A study published in *Science* found that an algorithm widely used to allocate healthcare resources among patients treated White and Black patients differently. Compared with White patients, Black patients were significantly less likely to be referred to a specialized care-management program for patients with complex medical needs, meaning that they often had to be more severely ill before receiving such medical support (27). It follows that the protection of personal health information is implicated not only in information leakage or privacy infringement, but also in the unfair analysis, classification, and use of information in algorithmic processing. Once such discriminatory processing enters the medical decision-making process, it may further translate into unequal opportunities to obtain medical resources, thereby impairing individuals’ equal health rights and interests.

Beyond discriminatory bias, algorithmic risks in medical AI also include inferential privacy harms, which further complicate the protection of personal health information and remain inadequately addressed by traditional legal frameworks (28). Traditional personal information protection regimes are primarily structured around stages such as the collection, storage, use, transmission, and disclosure of information, with their regulatory focus being placed on preventing personal information from being unlawfully obtained, disclosed, or processed beyond its authorized scope. In the medical AI context, however, the concern is not limited to the direct disclosure of existing information, but is more prominently manifested in processors’ use of algorithmic analytical capabilities to infer from existing health data sensitive information that the data subject has neither proactively provided nor expressly authorized for processing, such as disease predispositions, mental states, fertility risks, and future health trends. Although such algorithmically generated inferences are not mere reproductions of the contents of original medical records, their impact on private life, personal dignity, equality in employment and healthcare, and social evaluation is no less serious than the direct disclosure of existing health information.

Furthermore, medical artificial intelligence models themselves may become a new channel through which sensitive health information is exposed. Research in machine learning has shown that even where an attacker can access a model only in a black-box manner, it may still be possible to determine, through techniques such as membership inference, whether a particular individual’s information has been included in the training data (29). In medical settings, if a model is highly associated with a particular disease, treatment regimen, or patient group, once training-set membership is identified, it may further reveal a link between the individual and the corresponding sensitive health attribute, thereby giving rise to a substantive infringement of personal privacy and personality interests. The threat posed by medical artificial intelligence to personal health information has expanded from direct leakage at the database level to indirect exposure and associational identification in the processes of model training, algorithmic inference, and output generation.

## 5 Regulatory response for personal health information protection in the context of medical AI

### 5.1 Developing dedicated legislation for personal health information protection

As noted above, China has not yet enacted dedicated legislation specifically governing the protection of personal health information. Existing rules applicable to medical and health information remain dispersed across the Civil Code, the PIPL, the Data Security Law, and related regulatory instruments. While these norms provide a basic framework for personal information protection, they remain largely principled and abstract and therefore do not supply sufficiently specific standards for the processing of personal health information in concrete contexts. In the era of medical AI, where health data are increasingly aggregated, reused, and processed across multiple institutional and technical settings, such a fragmented framework can no longer provide protection that is both adequate and responsive to technological change. Accordingly, a more systematic and rigorous legal response is needed. Against this background, China should consider enacting a dedicated Personal Health Information Protection Law to strengthen the authority and practical effectiveness of the existing legal framework. Specifically, such legislation should address two fundamental issues: first, clarifying the scope of personal health information as the object of legal protection; and second, establishing specific legal bases and conditions for its processing.

#### 5.1.1 Clarifying the scope of personal health information

As the foundation of a dedicated legal framework, the first task of legislation is to determine the scope of information that should fall within the category of personal health information. In comparative legal practice, two primary terms are commonly used to describe health-related personal information or data: “health information” and “health data.” In substance, there is generally no fundamental difference between these two concepts, and the choice of terminology largely reflects legislative preferences and drafting conventions. European jurisdictions generally prefer the term “health data.” For example, the EU GDPR uses the expressions “data concerning health” in Article 4(15) and “personal data concerning health” in Recital 35 (30). By contrast, jurisdictions adopting a privacy-oriented regulatory approach tend to use the term “health information.” For example, the United States HIPAA adopts the concept of “individually identifiable health information” (31), while Canadian health privacy legislation, such as Ontario’s Personal Health Information Protection Act, refers to “personal health information” (32). Regardless of the terminology adopted, comparative legal frameworks generally understand health information or health data as information relating to the physical or mental health of an identified or identifiable individual. China currently lacks consistent terminology for health-related information or data. Future legislation should therefore adopt the unified concept of “personal health information” to enhance conceptual clarity and avoid unnecessary ambiguity.

With respect to how the scope of personal health information is delineated, comparative legal systems generally adopt three legislative approaches. The first approach defines the scope of health information through the enumeration of specific categories. HIPAA, for example, defines “individually identifiable health information” as information created or received by healthcare providers, health plans, employers, or healthcare clearinghouses that relates to an individual’s physical or mental health condition, healthcare services, or payment for healthcare services, and that identifies or can reasonably be used to identify the individual. Australia’s Privacy Act 1988 adopts a similar approach under section 6FA (33). The second approach relies on an abstract definition based on the general characteristics of health information. Under this model, the scope of health information is determined by describing the essential features of such information. For example, Article 4(15) of the GDPR defines “data concerning health” as personal data related to the physical or mental health of a natural person, including information revealing the health status of an individual through the provision of healthcare services. The third approach combines an abstract definition with a non-exhaustive enumeration of specific categories. This approach defines health information through general characteristics while also identifying representative types of information falling within the category. For example, Recital 35 of the GDPR adopts this approach by providing further clarification and examples regarding the scope of “personal data concerning health.” Each of these approaches has certain limitations. The abstract definition model may result in overly broad concepts that create uncertainty in legal application, whereas a purely enumerative model may lack sufficient flexibility to accommodate emerging technologies and evolving forms of health-data processing. To avoid these limitations, future Chinese legislation should adopt a combined approach that incorporates both a general definition and a non-exhaustive enumeration of representative categories when defining the scope of personal health information. Such an approach would provide greater legal certainty while maintaining sufficient flexibility to respond to future developments in medical artificial intelligence and health-data governance.

#### 5.1.2 Establishing specific legal bases for personal health information processing

Future legislation should clarify the circumstances in which consent is not required for the processing of personal health information and establish more detailed criteria governing the application of such exceptions. In addition to recognizing consent as an important legal basis for processing personal information, Article 13 of the PIPL identifies six circumstances in which personal information may be processed without consent and includes a residual clause for “other circumstances provided by laws and administrative regulations.” Although these provisions may also apply to certain personal health information processing activities, China’s current framework remains less specific and operationally effective than the more detailed exception mechanisms established under the EU and U. S. regulatory frameworks.

The HIPAA Privacy Rule establishes a differentiated framework governing the use and disclosure of protected health information according to different processing contexts and purposes. Specifically, 45 CFR §164.502(a)(1) establishes the general rule governing the use and disclosure of protected health information by covered entities, while specific permissible uses and disclosures are further regulated under subsequent provisions, including treatment, payment, healthcare operations, public health activities, and research-related exceptions. In the context of medical research, HIPAA further establishes substantive requirements for waiving or altering authorization requirements. Under 45 CFR § 164.512(i), an Institutional Review Board or Privacy Board may approve such a waiver or alteration only if the proposed use or disclosure involves no more than minimal risk to privacy, the research could not practicably be conducted without the waiver or alteration, and access to and use of protected health information are necessary for the conduct of the research. These requirements must also be supported by adequate safeguards to protect identifiers, plans for destroying identifiers at the earliest opportunity where appropriate, and written assurances limiting subsequent uses and disclosures. Similarly, Article 9 of the GDPR establishes a general prohibition on processing special categories of personal data, including data concerning health, while Article 9(2) sets out specific exceptions to this prohibition. For the processing of health-related data, the relevant exceptions primarily include explicit consent, substantial public interest, the provision of healthcare services, public health interests, and scientific research and statistical purposes. Compared with these frameworks, China’s PIPL does not currently provide equally specific and context-sensitive exceptions tailored to personal health information processing. Although Article 13(4) provides a legal basis for processing in response to public health emergencies or situations necessary to protect individuals’ life, health, and property, its application remains largely confined to emergency circumstances. Other provisions, such as Article 13(3) concerning the performance of statutory duties or obligations and Article 13(7) concerning other circumstances provided by laws and administrative regulations, may also serve as legal bases for certain personal health information processing activities; however, their application still requires further clarification through relevant legislation.

Accordingly, future legislation on personal health information protection in China should establish clearer legal bases for processing personal health information in contexts such as healthcare services, medical research, and public health governance. It should also specify the substantive conditions, procedural safeguards, and risk mitigation mechanisms applicable to such exceptions, thereby reducing regulatory uncertainty and enhancing the practical effectiveness of the legal framework.

### 5.2 Optimizing the consent model for personal health information processing

The PIPL recognizes individual consent as one of the important legal bases for processing personal information. Individuals must provide authorization voluntarily on the basis of sufficient information, thereby providing processors with a lawful basis for processing such information. In practice, however, consent often takes the form of static consent, referring primarily to one-time authorization granted at the initial stage of processing. This model is suitable for information processing activities where the processing purposes, methods, and participating entities remain relatively stable. Nevertheless, in the context of medical AI, the processing of personal health information is characterized by frequent processing activities, difficulties in specifying processing purposes in advance, and increasingly diverse processing contexts. Medical AI applications typically involve multiple stages of data utilization, including data collection, model training, algorithm optimization, and application expansion, during which the processing entities, contextual conditions, and potential risks may continue to evolve. Consequently, consent obtained at the initial stage may not adequately cover subsequent uses of health information, nor can it ensure that individuals maintain continuous awareness of and meaningful control over the flow and use of their health information.

To address the limitations of the traditional static consent model, it is necessary to develop consent mechanisms that correspond to the dynamic characteristics of medical AI data processing. In this regard, many Chinese scholars have proposed incorporating contextual integrity theory into consent regulation to better accommodate the evolving characteristics of information processing in the digital environment (34, 35). Contextual integrity theory recognizes that the appropriate level of protection and utilization of personal information varies across different contexts. Accordingly, the extent to which consent rules should regulate or facilitate information processing activities should be determined by the specific characteristics of each application context (36, 37). Under this theoretical framework, consent is no longer understood as an all-or-nothing choice, but rather as a flexible and evolving mechanism that can be adjusted according to contextual changes.

Building on this approach, scholars have proposed mechanisms such as tiered consent and dynamic consent to address the shortcomings of traditional consent models in complex data environments (38, 39). Tiered consent establishes differentiated consent requirements according to different processing contexts, creating a hierarchical framework ranging from strict authorization to broader forms of consent. By replacing the traditional bundled consent model, tiered consent enhances individuals’ ability to make selective choices regarding data processing and better reflects the normative objectives of informed consent. Dynamic consent, by contrast, addresses the limitations of one-time consent models by enabling individuals to maintain continuous awareness and decision-making control over the processing of their personal information through ongoing contextual risk assessment. It allows individuals to provide, modify, or withdraw consent when appropriate, thereby ensuring that information processing remains aligned with individuals’ reasonable expectations regarding the use of their data.

#### 5.2.1 Establishing a tiered consent mechanism based on information sensitivity and contextual risk

The current PIPL classifies medical and health information as sensitive personal information and uniformly applies the requirement of “separate consent” to such information. Although this approach reflects a strong protective orientation, it fails to adequately capture the varying levels of risk associated with different categories of health information. Particularly in the context of medical AI, where large-scale and multi-scenario data support is increasingly required, a uniform consent requirement may intensify “consent fatigue” and hinder the appropriate utilization of health data. Therefore, personal health information should be classified according to different levels of sensitivity, with corresponding tiered consent requirements established accordingly.

Tiered consent should be designed based on two primary factors: the sensitivity of personal health information and the context and associated risks of its use. Article 28(2) of the PIPL defines sensitive personal information based on the potential harm that may arise from unlawful disclosure or misuse, indicating that sensitivity classification should consider both the inherent sensitivity of the information and the severity of potential consequences. Accordingly, personal health information may be further categorized into highly sensitive, moderately sensitive, and less sensitivity information. The intensity of protection should correspond to the sensitivity level of health information. Information with higher sensitivity should be subject to more stringent protection requirements, whereas information with lower sensitivity may be governed by relatively less restrictive measures. In addition, because personal health information may be processed in diverse contexts, including clinical care, healthcare services, scientific research, and commercial applications, regulatory requirements should also take into account contextual risks. Since risks associated with health information processing may evolve when processing contexts change, contextual risk assessment should be designed as a dynamic mechanism.

Based on these considerations, consent requirements should be structured in a differentiated manner. For highly sensitive information involved in high-risk processing activities, more specific and explicit authorization should be required, together with enhanced prior notification and continuous control mechanisms. For example, when health information is used for new model training, cross-institutional sharing, automated decision-making, or the generation of significant health-related inferences, processors should reassess whether such processing exceeds individuals’ initial reasonable expectations and obtain renewed consent where necessary. By contrast, for processing activities where risks are relatively controllable, purposes are clearly defined, and relationships among relevant entities remain stable, tiered notification and scope-limited authorization may reduce unnecessary repeated consent requirements and mitigate consent fatigue. Such a differentiated approach avoids imposing excessive restrictions on all categories of health information while ensuring meaningful individual control over high-risk processing activities.

#### 5.2.2 Establishing a continuous disclosure and dynamic consent mechanism

Traditional informed consent is generally conceived as a one-time process, in which the signing of a consent form marks both the beginning and completion of the exercise of individual autonomy. However, such a model is increasingly inadequate for the era of medical AI, where personal health information is subject to frequent processing, repeated utilization, and expanding applications. Accordingly, informed consent should be transformed into a continuous mechanism through which disclosure obligations remain effective over time and consent can be updated in response to changes in processing activities.

Under the traditional one-time consent model, information disclosure primarily occurs at the initial stage of personal health information collection. Data subjects are typically informed only of the processing purposes and methods specified at that stage, while remaining unaware of subsequent uses of their health information, including AI model training, algorithm optimization, and other secondary applications. As a result, individuals may lose meaningful control over their health information after providing initial authorization, making it difficult to address the expanding scope of data utilization and evolving risks associated with medical AI. To overcome this limitation, processors should establish continuous disclosure mechanisms that enable data subjects to understand how their health information is being used throughout the processing lifecycle and make informed decisions based on changes in processing contexts and risk levels. Under such a mechanism, the processor’s duty of disclosure should not be exhausted after a single notification but should continue throughout the entire processing process. Similarly, consent should not be treated as a one-time authorization, but rather as an adjustable mechanism that allows individuals to modify the scope of permitted use, withdraw consent, and exercise ongoing control over their information.

At the operational level, processors should provide accessible technological tools that enable data subjects to review health information usage, manage authorization preferences, and update or withdraw consent. Both continuous disclosure and dynamic consent depend on effective communication mechanisms that facilitate ongoing interaction between processors and data subjects. With the advancement of digital technologies, electronic and network-based informed consent mechanisms provide the technical foundation for implementing dynamic consent. Through continuous information exchange and communication mechanisms, multi-stage disclosure and dynamic consent updating can be achieved throughout the processing lifecycle of personal health information, thereby protecting individual rights while supporting the responsible and sustainable development of medical AI.

### 5.3 Improving the whole-process regulation of medical AI algorithms

Beyond challenges concerning data processing, medical AI also generates new risks arising from algorithmic decision-making processes. Therefore, protecting personal health information requires not only regulating data processing activities but also ensuring accountability throughout the lifecycle of algorithmic systems. To mitigate the risks posed by medical artificial intelligence algorithms, a governance framework should be established that extends across the entire lifecycle of algorithm design, model training and ongoing operation (40).

At the design stage, transparency and explainability requirements should be built into the architecture of medical AI systems from the outset. It should be made clear which categories of information are subject to informed consent, the purposes for which they may be used, and the manner in which they will be collected and processed. The intended scope of algorithmic use, the basic logic of the system, and the conditions and limits of its clinical application should also be specified at the design stage, especially in relation to automated diagnosis, treatment recommendations, and other functions capable of materially affecting patients’ rights and interests (41). By embedding such requirements into system design, transparency becomes a structural feature of medical AI governance rather than a merely ex post obligation of disclosure.

At the training stage, targeted measures should be adopted to mitigate and, where feasible, eliminate algorithmic discrimination in medical AI. Such discrimination often arises from multiple sources, including data-driven bias, sample-selection bias, and inherent technical limitations. Developers should therefore collect data from diverse patient populations and take reasonable steps to improve representation across gender, race, and age groups, so that datasets more accurately reflect the populations to which the systems are intended to apply (42). In practice, the collection and recording of clinical data may be distorted by a range of factors, including human recording preferences, data-entry bias, and incomplete medical information, all of which may significantly affect downstream outputs. Strict data cleaning, validation, and standardization measures should therefore be required in order to identify and mitigate biases in the underlying data. The training stage should also be subject to appropriate documentation and audit requirements, so that the sources of training data, the methods of model optimisation, and the steps taken to reduce discriminatory bias remain open to regulatory scrutiny.

At the stage of deployment and ongoing operation, inferential privacy and model leakage should be expressly incorporated into the regulatory framework for medical AI. Medical AI may not only use existing health information to generate analytical judgments, but may also infer new health-related facts not voluntarily disclosed by individuals through multimodal association and pattern recognition, such as disease predispositions or future health trajectories. Although such algorithmically generated health information does not derive directly from original medical records, it may nonetheless infringe personal privacy and dignitary interests and therefore should not be excluded from the scope of health-information protection (43). At the same time, model-level risks such as membership inference and model inversion should be specifically addressed in order to prevent attackers from re-identifying training data through parameter behavior or predictive outputs (44). To this end, model security assessments should be conducted before deployment, with inferential privacy and model leakage expressly included within the scope of assessment, and model-level security governance should be strengthened accordingly. Once a system enters clinical use, controllers should remain under a continuing duty to monitor outputs, detect emergent bias and privacy risks, and make timely adjustments in light of feedback and changing clinical conditions. Where algorithmic updating results in a material change in the purposes of processing, the categories of information involved, or the functional scope of the system, renewed consent should be obtained pursuant to a dynamic consent mechanism, so as to prevent such updating from becoming a technical means of circumventing the original scope of authorization.

## 6 Conclusion

The rapid integration of artificial intelligence into healthcare has fundamentally reshaped how personal health information is generated, processed, and used. While medical AI offers significant benefits in improving the efficiency and quality of healthcare services, it also exposes structural limitations in existing legal frameworks for the protection of personal health information. As this article has shown, these limitations are most evident in the persistent tension between data protection and data utilization, the diminishing effectiveness of purpose limitation and informed consent in dynamic data-processing environments, and the growing complexity of algorithmic risks, including opacity, discrimination, and inferential privacy. To address these challenges, China needs to move beyond fragmented and abstract provisions and to establish a dedicated legal framework for personal health information. Such a framework should first clarify the concept and the scope of personal health information and establish context-specific rules governing its processing. In response to the limitations of traditional consent mechanisms in medical AI environments, China should establish a tiered consent mechanism based on the classification of personal health information and contextual risk assessment, complemented by continuous disclosure and dynamic consent mechanisms that enable individuals to maintain ongoing awareness and control over the use of their health information. In addition, the risks to personal health information arising from algorithmic systems should be governed through a whole-process framework for algorithmic governance. The experience of protecting personal health information in China not only reflects the challenges faced by jurisdictions undergoing rapid digital health transformation, but also highlights the importance of aligning legislation with technological change. The institutional responses and practical experience emerging from China may also offer useful lessons for other jurisdictions, particularly those undergoing digital healthcare transformation.

## Data Availability

The original contributions presented in the study are included in the article/supplementary material, further inquiries can be directed to the corresponding author.
